# Tumor in undescended intrapelvic testis revealed by supraclavicular lymphadenopathy: a case report and literature review

**DOI:** 10.1186/1756-0500-6-166

**Published:** 2013-04-26

**Authors:** Mohammed Fadl Tazi, Omar Riyach, Mustapha Ahsaini, Youness Ahallal, Abdelhak Khallouk, Mohammed Jamal El Fassi, Moulay Hassan Farih

**Affiliations:** 1 Department of urology, University Hospital Center Hassan II, Fes, Morocco; 2 Faculté de médecine et de pharmacie de Fès, BP : 1893 –Km 2.200 Route de Sidi Harazem, FES, Morocco

**Keywords:** Supraclavicular lymphadenopathy, Testicular tumor, Cryptorchidism

## Abstract

**Background:**

Testicular cancer is a rare disease. The incidence of testicular cancer in undescended testicles is of 3 to 48 times greater than in the general population. In the developed countries, the existence of undescended testicles in the adult population is rare, due to systematic practice of elective orchidopexy before the second year of life and orchiectomy in post adolescent males with undescended testicles. Despite these prevention measures, there are still some isolated cases of intra-abdominal testicular tumors in adults. We report a case of testicular cancer in cryptorchid testis revealed by supraclavicular lymphadenopathy.

**Case presentation:**

We report a case of a 46 year old fertile man with a history of unilateral cryptorchidism who presented with a palpable left supraclavicular mass and absence of the right testicle. On investigations an intrapelvic testis tumor was diagnosed. Laparotomy and complete excision was carried out. The possible association between the undescended testis and cancer transformations is briefly discussed.

**Conclusion:**

Testicular cancer in undescended testicles should not be ignored. Only early diagnosis and lower of testis in scrotumprevent such clinical forms.

## Background

Cryptorchidism (testicular maldescent), the most common congenital anomaly of the genitourinary tract in males, is encountered in 1% of boys. The incidence increases in subjects with deficiencies of androgen function [1]. Such an organ is at high risk of torsion, trauma, infertility, and malignancy. A tumor of an intra-pelvic testis in a fertile patient revealed by supraclavicular lymphadenopathy is reported in this case.

## Case presentation

**Figure 1:**
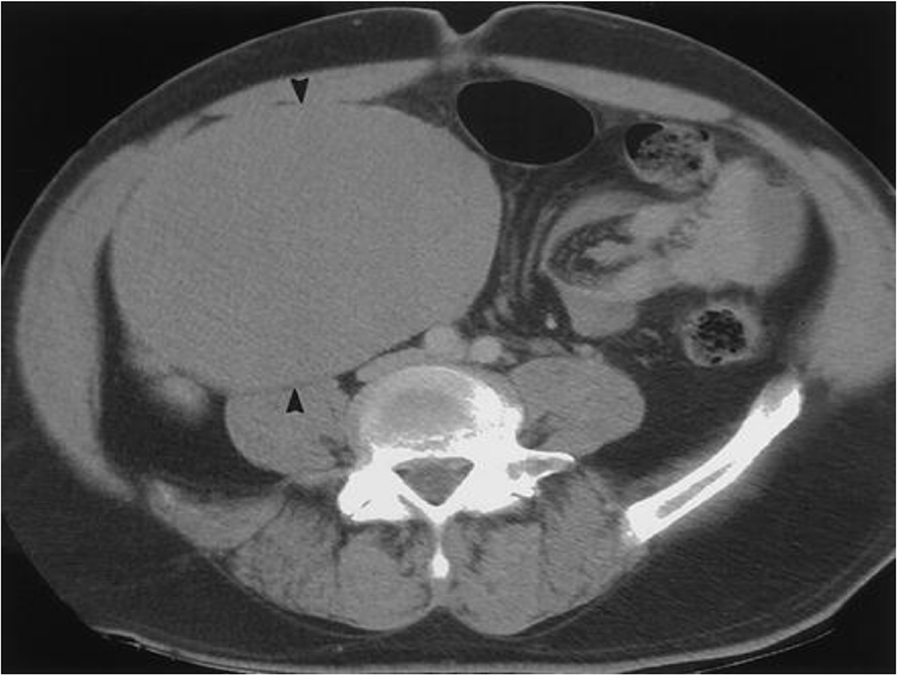
CT image of the abdomen shows a large retroperitoneal pelvic mass.

**Figure 2:**
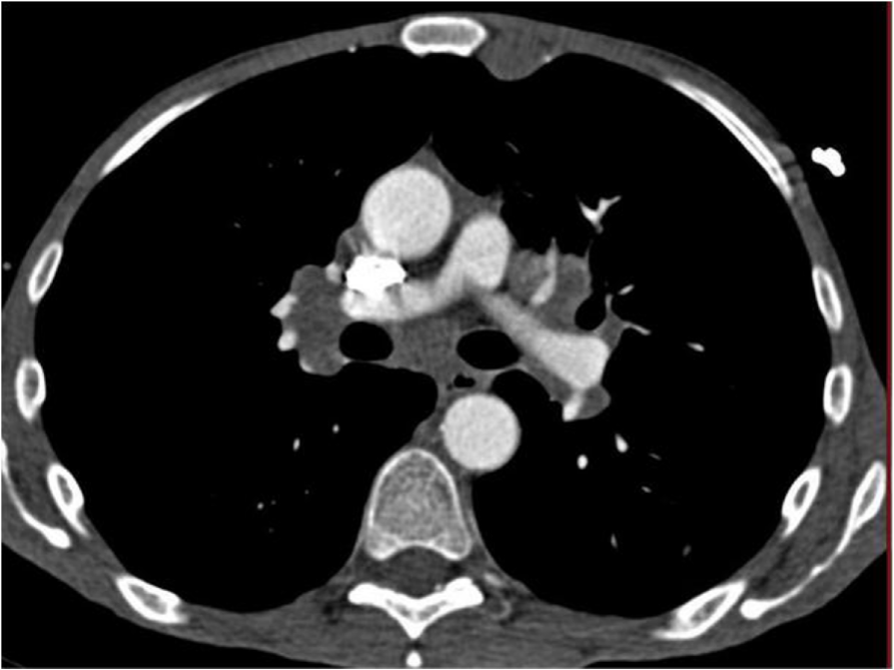
CT image of the thorax shows mediastinal lymph nodes.

A 46 year old man, father of three children, without significant previous medical history, consulted for the gradual emergence of an isolated left supraclavicular mass. On physical examination, the patient was afebrile, in good general condition, pleuropulmonary and abdominal examination was normal. The genital examination found an empty right purse, the left testicle was normal. The inguinal examination found no palpable mass but a suspect induration was reported. The ORL examination revealed no abnormalities. Chest radiography showed a normal pulmonary transparency. On ultrasonographic scanning of supraclavicular fossa and whole abdomen showed a supraclavicular lymphadenopathy measuring 31 × 23 mm and an heterogeneous mass in the right iliac fossa with irregular contours. The laboratory investigations were all normal; apart from a slightly elevated serum beta- human chorionic gonadotropin (β-HCG) 428 mmol/L. The serum alpha- fetoprotein (α-FP) was within normal limits, and lactate dehydrogenase (LDH) at 308 U/l. The thoracoabdominopelvien computed tomography (CT) scan revealed a right pelvic mass measuring 100 × 80 mm (Figure 1), and mediastinal lymph nodes (Figure 2). No intraperitoneal or retroperitoneal abnormalities were noted. A biopsy of supraclavicular node was performed and revealed metastatic lymphadenopathy of an embryonal carcinoma. The patient underwent a laparotomy that confirmed the diagnosis of an intra-abdominal testicular tumor. The resection was performed without complications. The pathological analysis revealed a non-seminomatous tumor. The postoperative tumor markers were within normal stage. The patient was referred to receive adjuvant chemotherapy consisting of a combination of cisplatinum 20 mg/m2, etoposide 100 mg/m2 given on five consecutive days and bleomycin 30 mg on days one, eight, and 15. There was no clinical evidence of local recurrence or distant metastasis after 18-months follow up after discharge.

## Discussion

The testicular cancer represents 1-2% of all male malignant tumors and 4% of the urogenital ones. It is the more frequent solid neoplasia in young men between 20-35 years meaning the 1-2% of total tumors [1] There are many etiological proposed factors in the development of testicular cancer: traumatisms, testicular atrophy, and gonadal dygenesis, being cryptorchid and the history of tumor in the contralateral testicle the most significant [2,3]. In cryptorchidic testicles the incidence of testicular cancer is considered between 3 to 48 times greater than in the general population [4,5]. Approximately a 10% of all the testicular tumors appear on an undescendent testicle [4,5].

Cryptorchid affects 0, 4% of male. The non-palpable testicles correspond to a 20% of the cryptorchidic testicles. Of the non-palpable testicles, only in 20% of the cases it is absent, the rest is in the abdomen or the inguinal canal. Between undescended testicles, abdominal testicles present a higher rate of malignity than the ones located in the groin inguinal [4]. The abdominal testicles develop cancer in 30% of cases [6].

The tumors in undescendent testicles are rare. The histopathology of the undescendent testicle tumors in the adult depends on location, being the proportion of pure seminoma of 93% when it is in intra-abdominal situation, 63% if it is inguinal and 28% in normotopic testes [7]. The prognosis will depend on initial stage and tumor histology.

In the developed countries, the existence of undescended testicles in the adult population is rare, due to systematic practice of elective orchidopexy before the second year of life, to prevent cancer and infertility. Orchidopexy does not eliminate cancer risk but allows an early diagnosis being testicle accessible to exploration [8,9].

There are many studies published on cryptorchidism and cancer in undescendent testicles. There is no long series of intra-abdominal testicular tumors in the literature. The majority of them corresponds to a case reports. Searching in PubMed, they are approximately 42 clinical cases of seminomas in intra-abdominal testicles published. In a 1930 article, a 8% incidence of Supra-calavicular lymphnodular metastasis have been recorded. In an article from Kenyata, an incidence of 17.91% has been recorded [10]. The greatest part was between the decade of 50 and 80. Afterwards, there were less published cases due to advances in investigation on the evolution of undescendent testicles, its relation with cancer and the prevention actions taken. In the last 20 years there were less published cases, which would be explained by the progressive reduction due to the prevention of tumors incidence in abdominal testicles [11].

## Conclusion

The abdominal variant of cryptorchid testis is rare and carries a high risk of malignant transformation. Very rarely they can be revealed by a supra-calavicular node. Early diagnosis and treatment of cryptorchidism can dramatically reduce the risk of testicular cancer and probably preserve the fertility, as well as, the virility of the patient.

## Consent

Written informed consent was obtained from the patient for publication of this manuscript and accompanying images. A copy of the written consent is available for review by the Editor-in-Chief of this journal.

## Competing interests

Authors declare that they have no competing interests.

## Authors’ contributions

MFT, OR, MA: the principal authors, major contributions in writing the manuscript. YA, AK, MJE, and MHF: analysed and interpreted the patient data and the reviews of the literature. All authors read and approved the final manuscript.
